# Cerebellar Morphology and Behavioral Profiles in Mice Lacking Heparan Sulfate *Ndst* Gene Function

**DOI:** 10.3390/jdb8030013

**Published:** 2020-07-11

**Authors:** Lars Lewejohann, Srinivas R. Pallerla, Rebecca S. Schreiber, Joanna Gerula, Kay Grobe

**Affiliations:** 1 Department of Behavioral Biology, Westfälische Wilhelms-Universität Münster, D-48149 Münster, Germany; Lars.Lewejohann@fu-berlin.de (L.L.); rebecca.schreiber@uni-muenster.de (R.S.S.); jgerula@uni-muenster.de (J.G.); 2 Institute of Tropical Medicine, University of Tübingen, 72074 Tübingen, Germany; srinivas-reddy.pallerla@uni-tuebingen.de; 3 Institute of Physiological Chemistry and Pathobiochemistry, Westfälische Wilhelms-Universität Münster, 48149 Münster, Germany

**Keywords:** Purkinje cell, heparan sulfate, extracellular matrix, knockout mouse, Ndst

## Abstract

Disruption of the Heparan sulfate (HS)-biosynthetic gene N-acetylglucosamine N-Deacetylase/N-sulfotransferase 1 (*Ndst1*) during nervous system development causes malformations that are composites of those caused by mutations of multiple HS binding growth factors and morphogens. However, the role of *Ndst* function in adult brain physiology is less explored. Therefore, we generated mice bearing a Purkinje-cell-specific deletion in *Ndst1* gene function by using Cre/loxP technology under the control of the Purkinje cell protein 2 (Pcp2/L7) promotor, which results in HS undersulfation. We observed that mutant mice did not show overt changes in the density or organization of Purkinje cells in the adult cerebellum, and behavioral tests also demonstrated normal cerebellar function. This suggested that postnatal Purkinje cell development and homeostasis are independent of *Ndst1* function, or that impaired HS sulfation upon deletion of Ndst1 function may be compensated for by other Purkinje cell-expressed Ndst isoforms. To test the latter possibility, we additionally deleted the second Purkinje-cell expressed Ndst family member, Ndst2. This selectively abolished reproductive capacity of compound mutant female, but not male, mice, suggesting that ovulation, gestation, or female reproductive behavior specifically depends on Ndst-dependent HS sulfation in cells types that express Cre under Pcp2/L7 promotor control.

## 1. Introduction

Heparan sulfate (HS) is produced by most mammalian cells as part of membrane and extracellular matrix proteoglycans [1]. The chain grows by exostosin (Ext) copolymerization of GlcAβ1,4 and GlcNAcα1,4 and is modified by one or more of the four Ndst isozymes; the N-deacetylase activity of Ndsts removes acetyl groups from GlcNAc residues, which are then converted to GlcNS through the N-sulfotransferase activity. Subsequent modifications of the HS chain by most O-sulfotransferases and a GlcA C5-epimerase depend on the presence of GlcNS residues, making the Ndsts responsible for the generation of sulfated ligand binding sites in HS [2]. *Ndst1* and *Ndst2* mRNA are expressed in all embryonic and adult tissues examined, whereas *Ndst3* and *Ndst4* transcripts are predominantly expressed during embryonic development [3].

Many growth and survival factors bind to HS, and HS-proteoglycans often act as co-receptors for these ligands. Studies in *Drosophila melanogaster* demonstrated that HS is crucial for embryonic development [4] and that the fly Ndst ortholog, Sulfateless, affects signaling mediated by Wingless (Wg), Hedgehog (Hh) and Fibroblast growth factor (Fgf) [5,6,7]. Because those factors also play critical roles in vertebrate morphogenesis, growth regulation and differentiation, defective HS synthesis affects multiple aspects of vertebrate development as well. Mice deficient in *Ext*1, *Ndst1*, *2-Ost* and *GlcA C5-epimerase* gene functions show defective brain morphogenesis, axon guidance defects, craniofacial defects, renal agenesis and eye defects due to the simultaneous inhibition of multiple HS binding factors during development [8,9,10,11,12,13]. Targeted deletion of HS biosynthesis in the nervous system of adult mice results in behavioral defects, including autism-like socio-communicative disorders in conditional HS-mutants [14] and the BTBR T + tf/J mouse model [15]. Defects in HS modification in the adult nervous system have further been found to affect spatial learning and cage activity of mice [16], and changes in the expression of heparan sulfate proteoglycans syndecan 3, syndecan 1, and heparanase (a HS degrading enzyme) lead to behavioral changes, such as increased or decreased food intake and enhanced LTP and impaired hippocampus-dependent memory [17,18,19,20]. Notably, HS sulfation changes upon deletion of the *Ndst3* gene have also been linked to changes in anxiety-related behavior in mice [21], and mutations of the same gene in humans have recently been linked to schizophrenia and bipolar disorder [22], possibly due to the impaired function of unknown HS-binding factors.

HS, as well as numerous HS-binding neurotrophic and neuroprotective factors, are expressed in the adult brain, including but not restricted to fibroblast growth factor 2 and 8 (Fgf2, Fgf8), Sonic Hedgehog (Shh), Neurotrophins, Insulin-Like Growth Factor I (Igf-1), epidermal growth factor (Egf) and glia-derived neurotrophic factor (Gdnf) [23,24,25,26,27,28,29,30,31,32,33]. Impaired function of those factors or their receptors leads to progressive neurodegeneration [34,35,36,37], mainly due to increased apoptosis that was also observed in the developing nervous system of Ndst1-deficient embryos [9]. Thus, impaired HS sulfation may affect brain homeostasis and function, as previously suggested [38,39,40,41,42].

We tested this possibility using the cerebellum as a model. This is because the cerebellum is relatively simple, consisting of only five types of neurons: Purkinje cells, granule cells and three types of interneurons. Cerebellar defects result in ataxia (failure of muscular coordination) that can be readily observed. In addition, the cerebellum plays an important part in the coordination of behavior. For those reasons, and because we found Ndst1 to be strongly expressed in this cell type, we generated mice bearing a Purkinje cell-specific deletion in the HS biosynthetic enzyme Ndst1. In this work, we describe that the morphology of the brain in conditional *Ndst1*-mutant mice was largely unaffected, consistent with data from another HS-deficient mouse model [16], and that only subtle locomotor and coordination as well as behavioral defects were observed. This indicates non-essential Ndst1 functions or compensation of loss of Ndst1 function by other expressed Ndst isoforms in Purkinje cells. Consistent with the latter possibility, we describe that additional systemic deletion of Ndst2 resulted in poor reproductive capabilities of compound mutant female mice. This suggests that combined loss of Ndst1 and Ndst2 protein function and resulting HS undersulfation in cells expressing Cre recombinase under the control of the Pcp2/L7 promotor strongly impairs female reproductive behavior.

## 2. Materials and Methods

**Figure 1:**
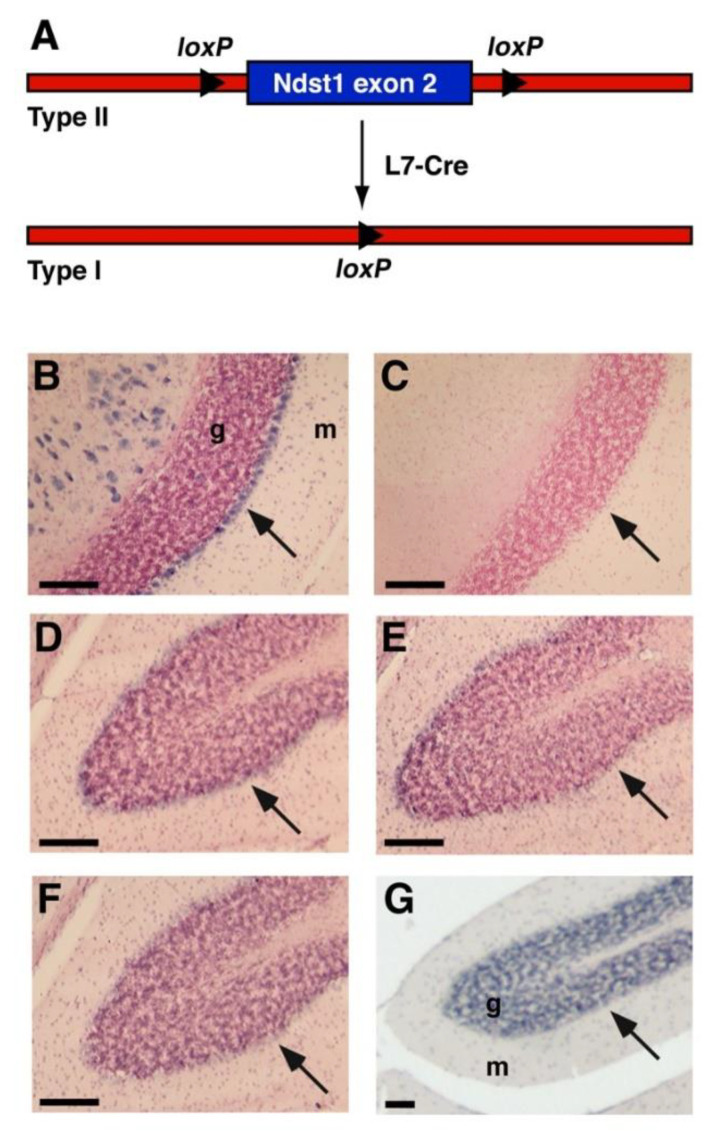
Disruption of the *Ndst1* gene expressed in cerebellar Purkinje neurons by targeted recombination. (**A**) Maps of the Type II “floxed” *Ndst1* allele and a cell-type-specific Type I deletion allele, obtained after breeding of Type II mice with *L7-Cre* mice. Lox-sites located in intron sequences are shown as triangles. (**B**–**G**) In situ hybridization of adult wildtype cerebellum showed strongest *Ndst1* expression (B, blue stain) in cerebellar Purkinje cells (arrows) as well as in unidentified cell types in the white matter, possibly myelin-forming oligodendroglia. Sense controls showed no reactivity (**C**). *Ndst2* (**D**), *Ndst3* (**E**) and *Ndst4* (**F**) expression were barely detectable in Purkinje cells. Tissue sections were probed with an antisense riboprobe specific for *Ndst1-4*. (**G**) H&E staining of the corresponding cerebellar area. g: granule cell layer, m: molecular layer. Scale bars: 100 μm.

*Mice.* The generation of the *Ndst1^f^* allele has been described previously [9]. Breeding with mice expressing Cre-recombinase under the control of Purkinje cell protein 2 (Pcp2 or L7) resulted in specific, postnatal Ndst1 deletion in cerebellar Purkinje cells [43] (Figure 1A).

*Behavior:* For behavioral tests, male and female wildtype (*n* = 12, 9), heterozygous (*n* = 13, 11) and mutant mice (*n* = 9, 13) were compared. Mice were kept under a 12 h/12 h light dark cycle for 3 weeks before testing began. Food (10 H 10, Nohrlin GmbH, Bad Salzuflen, Germany) and water were available ad libitum. Mice were housed in standard (37 (l) × 21 (w) × 15 (h) cm) cages, with up to five sex-matched animals per cage. Ear punctures allowed the discrimination of individual mice but behavioral experiments were carried out with the experimenter being unaware of the genotypes of the subjects. The cages contained a thin layer of wood shavings and paper towels as nesting material. Mice were looked after daily, but handled only once a week while transferring them to clean cages. All procedures and protocols met the guidelines for animal care and experiments in accordance with national (Animal research permit number A32/2006) and European (86/609/EEC) legislation. General health and neurological status were assessed using a protocol including tests as described elsewhere [44]. Animals were inspected for physical appearance and underwent neurological testing including acoustic startle, visual placing, grip strength and reflex functions to ensure that behavioral findings were not the result of deteriorating physical conditions of the animals. The Rotarod test was employed to assess balance and motor function (TSE RotaRod, TSE Systems GmbH, Bad Homburg, Germany). Mice were placed on the rotating cylinder of 3 cm diameter, and latency to fall off the rotating cylinder was measured automatically. Initial speed set to 4 rpm was accelerated to 400 rpm within a maximum session time of 300 s. Mice conducted two trials per day on 5 consecutive days. In the open-field test mice had the opportunity to explore a square arena (80 by 80 cm, walls 40 cm high) for 5 min. Locomotor activity and the ratio between exploration and fear of open space, as measured by the time spent near the walls or in the center of the arena, were assessed automatically by a tracking system [Lewejohann, L. Digital Image Processing in Behavioral Sciences. http://www.phenotyping.com/digital-image-processing.html. Date of Access: 03/07/2020]. In the Dark–Light test, anxiety-related behavior, e.g., the tendency of mice to stay in dark sheltered areas, was measured. The test apparatus consisted of a standard cage with one-third of the area being painted in black and covered by an opaque plastic ceiling. The dark compartment was separated from the light compartment by an opaque wall with a guillotine door. Each mouse was placed into the dark compartment for 1 min before the door was opened. Latency to enter the light compartment as well as the time spent in the light compartment were measured for 5 min using a Palm handheld computer and proprietary software for behavioral recording. Anxiety-related behavior was further assessed using the Elevated plus-maze [45]. Here, mice had the choice of moving into opposing arms, which were either shielded or open. The maze was elevated 50 cm above the floor and arms were 30 cm long and 5 cm wide. At the beginning of a trial, mice were placed into the center of the maze, facing one of the shielded arms. Each entry into an open or shielded arm was counted and the time animals spent in either type of arm was measured automatically using a tracking system [Lewejohann, L. Digital Image Processing in Behavioral Sciences. http://www.phenotyping.com/digital-image-processing.html. Date of Access: 03/07/2020]. Data analysis was conducted using the statistical software “R” (http://www.r-project.org/). Pairwise comparison of datasets that showed a non-Gaussian distribution was done two-tailed using the two-sample Wilcoxon test. A repeated-measures ANOVA was calculated to analyze performance over time in the Rotarod. The Chi2 test was used to determine statistical significance between litter sizes of *Ndst1*;*Ndst2* compound mutant mice. A *p*-value <0.05 was considered as statistically significant.

*Histology and detection of mRNA expression.* Mouse brains were fixed in 4% paraformaldehyde overnight, dehydrated, embedded in paraffin and sectioned at 8 μm. Sections were stained with hematoxylin and eosin for histological analysis. For in situ hybridization, 700 base probes against the most variable N-terminal regions of *Ndst1* and *Ndst4*, or directed against the 3′UTR of *Ndst2* and the 5′UTR of *Ndst3* were employed (DIG RNA Labeling Kit, Roche, Mannheim, Germany).

Adult brains were dissected and fixed in 4% paraformaldehyde solution overnight, dehydrated, embedded in paraffin and sectioned. For immunohistochemical analysis, the 10 µm paraffin sections were deparaffinized using xylol and rehydrated by a series of Ethanol/PBS washes. Epitope retrieval was done by boiling slides by using microwave in 0.1 M sodium citrate, pH 6.0, for 20 min, followed by three washing steps in PBS. Blocking and antibody incubation of the sections was done in 1X PBS, 5% BSA, 0.05% Tween 20. When HRP secondary antibody was used, sections were pretreated with 0.3% H_2_0_2_ in methanol to quench endogenous peroxidases. Immunohistochemical Ndst1 analysis was performed using α-Ndst1 antisera (1:50) (kindly provided by Dr. Lena Kjellen, Uppsala, Sweden), followed by detection using Alexa-labelled α-IgG H + L antibody (1:200, Molecular Probes, Eugene, OR, USA). Sections were incubated overnight with α-calbindin antibodies (1:200, Acris antibodies GmbH, Hiddenhausen, Germany) at 4 °C, and detected using secondary antibodies α-IgG H + L antibody (Alexa-labelled, 1:200, Molecular Probes, Eugene, OR, USA) or α-mouse HRP (1:500, Dianova, Germany). For detection of Cre antigen, sections were incubated with polyclonal α-Cre recombinase antibody (1:1000, Novagen, Germany) overnight at 4 °C, followed by incubation with biotinylated secondary anti-rabbit antibodies (1:150, Dianova, Germany) and streptavidin-HRP (1:2000, Dianova, Germany).

## 3. Results

### 3.1. Ndst1 Expression in the Mouse Cerebellum

Aberrant sulfation of HS upon Ndst1 deletion in mice affects several neurodevelopmental processes and is perinatal/postnatal lethal [9,10,12,13,21]. To study the roles of HS in adult brain homeostasis in vivo, mice harboring a conditional, loxP-flanked allele of *Ndst1* (*Ndst1^f/f^*) were thus crossed with transgenic mice expressing the bacteriophage recombinase Cre under the control of the Purkinje cell protein 2 (Pcp-2 or L7) promotor, which is predominantly expressed in postnatal cerebellar Purkinje cells [43] (Figure 1A). Postnatal (P30) Purkinje cells in the cerebellum strongly express *Ndst1*, and only limited amounts of *Ndst2*, as demonstrated by in situ hybridization (Figure 1B,D). *Ndst3* and *Ndst4* expression was weak or absent (Figure 1E,F). This identifies Purkinje neurons are the main HS-producing cell type in this tissue, making them ideal to study the role of neuronal HS in adult brain function. To this end, the well-established method of cell-type specific Cre-mediated Ndst1 deletion was employed [9,46,47,48].

### 3.2. Cerebellar Architecture after L7 Mediated Ndst1 Deletion

**Figure 2:**
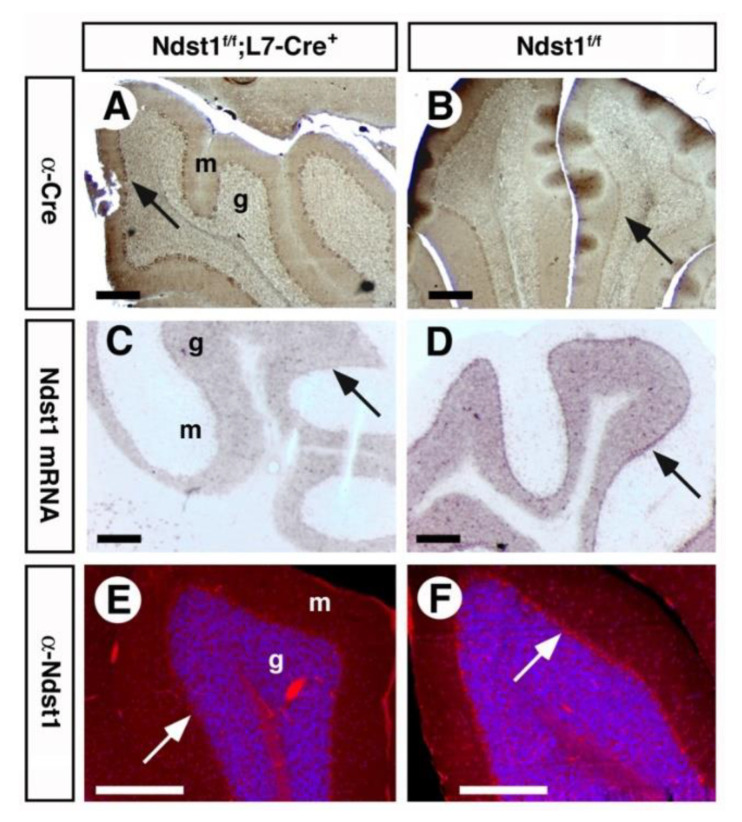
*Ndst1* mRNA and protein expression is abolished in Cre-expressing Purkinje cells of *Ndst1^f/f^;L7-Cre^+^* mice. (**A**) Immunohistochemical detection of Cre expression (brown stain) in adult cerebellar sections. Cre expression restricted to the Purkinje cell layer of *L7-Cre^+^* mice (arrow) but absent from mice carrying the floxed allele of Ndst1 (**B**). In situ hybridization employing an antisense probe directed against exon 2 of Ndst1 mRNA demonstrates loss of the floxed sequence in *L7-Cre^+^* transgenic mice (arrow) but not in Type *2 Ndst1* transgenes (**C**,**D**), and immunohistological detection of Ndst1 protein using the affinity-purified rabbit-anti-mouse Ndst1 antiserum demonstrates loss of the protein only in targeted cells of *Ndst1^f/f^;L7-Cre^+^* transgenic mice (**E**,**F**, arrow). g: granule cell layer, m: molecular cell layer. Scale bars: 200 μm.

Next, we stained cerebellar sections of *Ndst1^f/f^;L7-Cre^+^* (mutant) and *Ndst1^f/f^* (producing wildtype HS) mice with anti-Cre antibodies to confirm specific expression of the recombinase in cerebellar Purkinje cells (Figure 2A). L7/pcp expresses Cre-recombinase starting from postnatal day (P)6, with strongest expression from P14 to P21 [43]. Consistent with this, we detected Cre-expression in *Ndst1^f/f^;L7-Cre^+^* mice at P20 and throughout adulthood. Thus, Ndst1 function is deleted during the time when murine cerebellar anatomy, synaptic connectivity and neuronal morphology are established (up to the third to fourth postnatal week). Indeed, in situ hybridization employing a 500 bp probe directed against the floxed exon 2 confirmed Ndst1 deletion in Cre-expressing Purkinje cells (Figure 2C). Loss of Ndst1 protein in Cre-expressing double transgenic Purkinje cells of *Ndst1^f/f^;L7-Cre^+^* mice, but not in *Ndst1^f/f^* mice, was further confirmed immunohistochemically (Figure 2E).

Consistent with postnatal onset of Cre-expression, *Ndst1^f/f^;L7-Cre*^+^, *Ndst1^+/f^;L7-Cre^+^* and *Ndst1^+/+^;L7-Cre^+^* mice were born at the expected mendelian ratio. We observed that at 6 weeks, mice were of normal weight (female *Ndst1^f/f^;L7-Cre^+^*: 22,4 g ± 1 g versus *Ndst1^+/+^;L7-Cre^+^*: 22.2 g ± 1 g, *p* > 0,68, *n* = 21; male *Ndst1^f/f^;L7-Cre^+^*; 29 g ± 3 g versus *Ndst1^+/+^;L7-Cre*^+^: 30,7 g ± 2 g, *p* > 0,2, *n* = 21), indicating that the feeding behavior of conditional *Ndst1* mutant mice was not altered. Moreover, adult mutant mice were indistinguishable from wild-type littermates in general health and reproduced normally, indicating that postnatal deletion of Ndst1 in Purkinje cells did not result in obvious defects.

**Figure 3:**
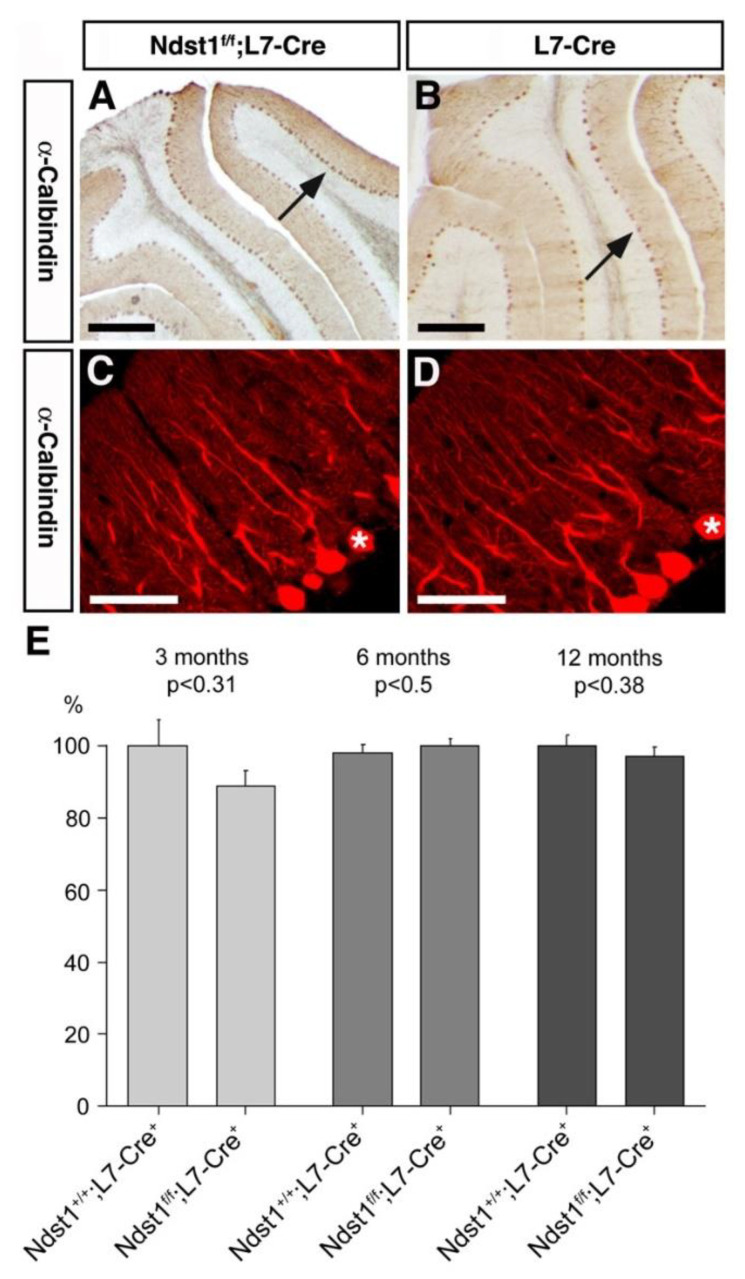
Cerebellar morphology and Purkinje cell number and morphology are unchanged in *Ndst1^f/f^;L7-Cre^+^* mutant mice. Calbindin staining reveals similar cerebellar morphology and Purkinje cell numbers when comparing (**A**) *Ndst1^f/f^;L7-Cre^+^* and (**B**) *L7-Cre^+^* mice. Scale bars: 200 μm. Purkinje cell morphology is also unaltered (**C**,**D**). Scale bars: 50 μm. (**E**): Purkinje cell number is unaffected after 3 months, 6 months and 12 months. Cells were counted and plotted as relative % of the control number.

Therefore, we next employed immunohistochemistry using anti-calbindin antibodies to assess possible changes in the number and morphology of Purkinje cells. However, no differences in cerebellar folia development, laminar organization or cross-sectional areas of the molecular or granule cell layers were found between control and *Ndst1^f/f^;L7-Cre^+^* mice, and no significant changes in the number, density or organization of Purkinje cells was observed. This suggested that survival of these projecting neurons in vivo does not require Ndst1 function (Figure 3A,B). Calbindin staining also showed that primary and secondary trees of Purkinje cells were present and similarly branched in *Ndst1^f/f^;L7-Cre^+^* conditional mutant mice and *Ndst1^+/+^;L7-Cre^+^* controls (Figure 3C,D). Lastly, the number of Purkinje cells was determined in 3 months, 6 months and 12 months old mice to assess possible neuronal cell death. Again, no significant differences were detected between *Ndst1^f/f^;L7-Cre^+^* and *Ndst1^+/+^;L7-Cre^+^* control mice (Figure 3E).

### 3.3. Normal Motor Function in Ndst1^f/f^;L7-Cre^+^ Mutant Mice

**Figure 4:**
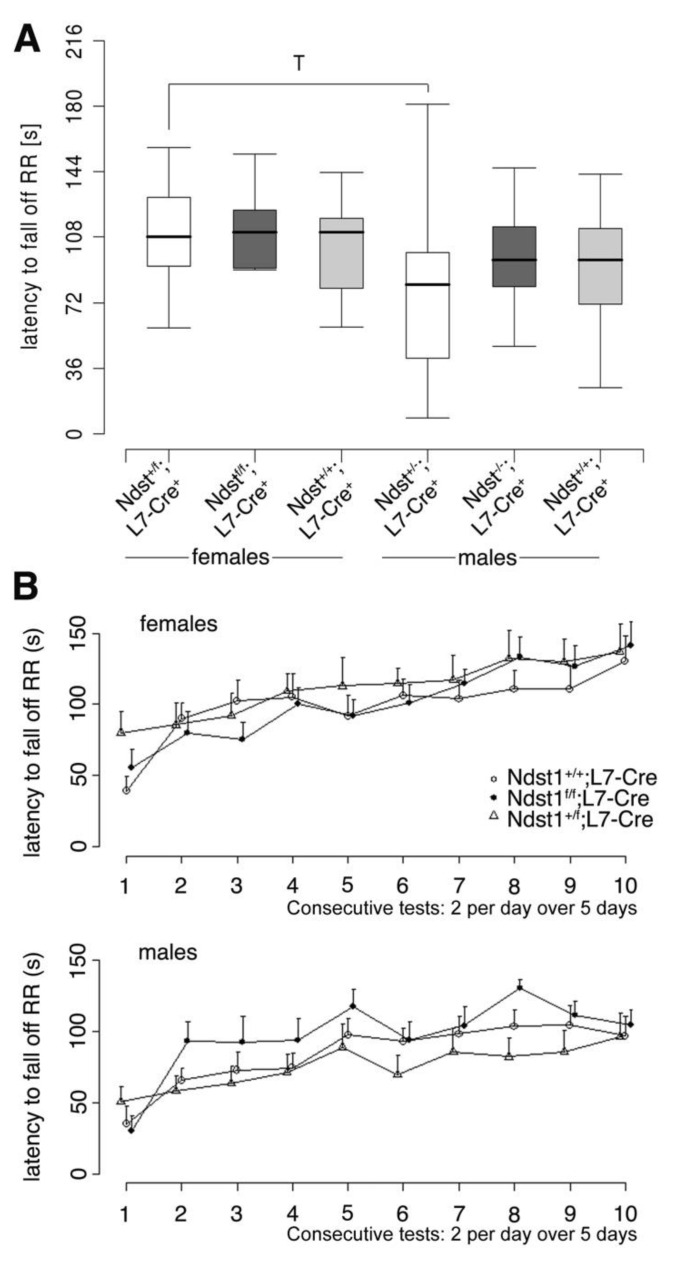
Locomotor abilities and balance are normal in Ndst1 mutant mice. (**A**) Latencies to fall off the Rotarod were measured in 10 trials on 5 consecutive days. (**B**) Repeated-measures using ANOVA with genotype and sex as independent measures and trials as repeated factors revealed significant sex differences (F(1580) = 29.8; *p* < 0.001) and an overall improvement over trials (F(9580) = 10.9; *p* < 0.001) but no effect of genotype. f *Ndst1^+/f^;L7-Cre^+^ n* = 11, f *Ndst1^f/f^;L7-Cre^+^ n* = 13, f *Ndst1^+/+^;L7-Cre^+^ n* = 9, m *Ndst1^+/f^;L7-Cre^+^ n* = 13, *m Ndst1^f/f^;L7-Cre^+^ n* = 9, m *Ndst1^+/+^;L7-Cre^+^ n* = 12.

Because no histological abnormalities were found upon Purkinje-cell specific Ndst1 deletion, we next assessed motor function of *Ndst1^f/f^;L7-Cre^+^* mutant mice and controls as a functional read-out. Mice underwent a general health check to rule out that differences observed were due to deteriorating health of the mice, and the latency to fall off a Rotarod was used as a standard measure of motor function and coordination. Again, we found that Rotarod performance did not differ significantly between conditional *Ndst1^f/f^;L7-Cre^+^* mutant and *Ndst1^+/+^;L7-Cre^+^* control mice (Figure 4A). Both male and female groups showed improved performance over 10 trials (F(9580) = 10.9; *p* < 0.001) (Figure 4B). ANOVA further revealed a significant effect of sex (F(1580) = 29.8; *p* < 0.001) but genotype differences were not significant (F(2580) = 1.26; *p* > 0.28), indicating that no functional deficiencies resulted from Ndst1 deletion in Purkinje cells in the adult brain.

### 3.4. Analysis of Ndst1 Expression and Function in the Adult Brain

**Figure 5:**
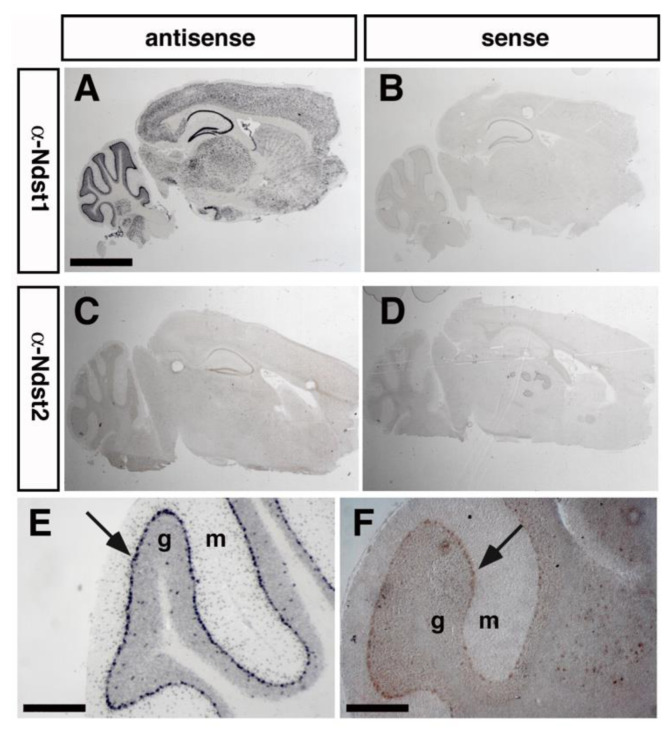
Widespread expression of *Ndst1* in the adult brain, and partially overlapping *Ndst2* expression in cerebellar Purkinje cells, cortex and hypothalamus. (**A**) *Ndst1* mRNA is widely expressed in various brain areas, notably the hippocampus and the cerebellum. Scale bar: 2 mm. (**B**) sense control mRNA demonstrates specificity. (**C**) *Ndst2* expression throughout the adult brain. (**D**) sense control mRNA. (**E**) In situ hybridization analysis demonstrates strong *Ndst1* expression in cerebellar Purkinje cells, and weak but overlapping expression of the *Ndst2* isoform (**F**). Scale bar: 200 μm. m: molecular layer, g: granule cell layer.

*L7-Cre* is also expressed in various brain areas outside of the cerebellum, such as the olfactory bulb, cerebral cortex, caudate putamen, hippocampus, thalamus, midbrain and medulla oblongata [49]. Here (Figure 5A,C), as well as in Purkinje neurons (Figure 5E,F), robust *Ndst1* and *Ndst2* expression were detected, indicating that tissues other than the cerebellum may be functionally affected in *Ndst1^f/f^;L7-Cre^+^* mice. Because Ndst3-deficient mice have been reported to show subtle changes in anxiety-like behavior [21], and because *Ndst3* mutations have been linked to shizophrenia and bipolar disorder in humans [22] that both include anxiety as a symptom, we next assessed anxiety, anxiety-related behavior and exploratory behavior in *Ndst1^f/f^;L7-Cre^+^* mice and *Ndst1^+/+^;L7-Cre^+^* control mice.

**Figure 6:**
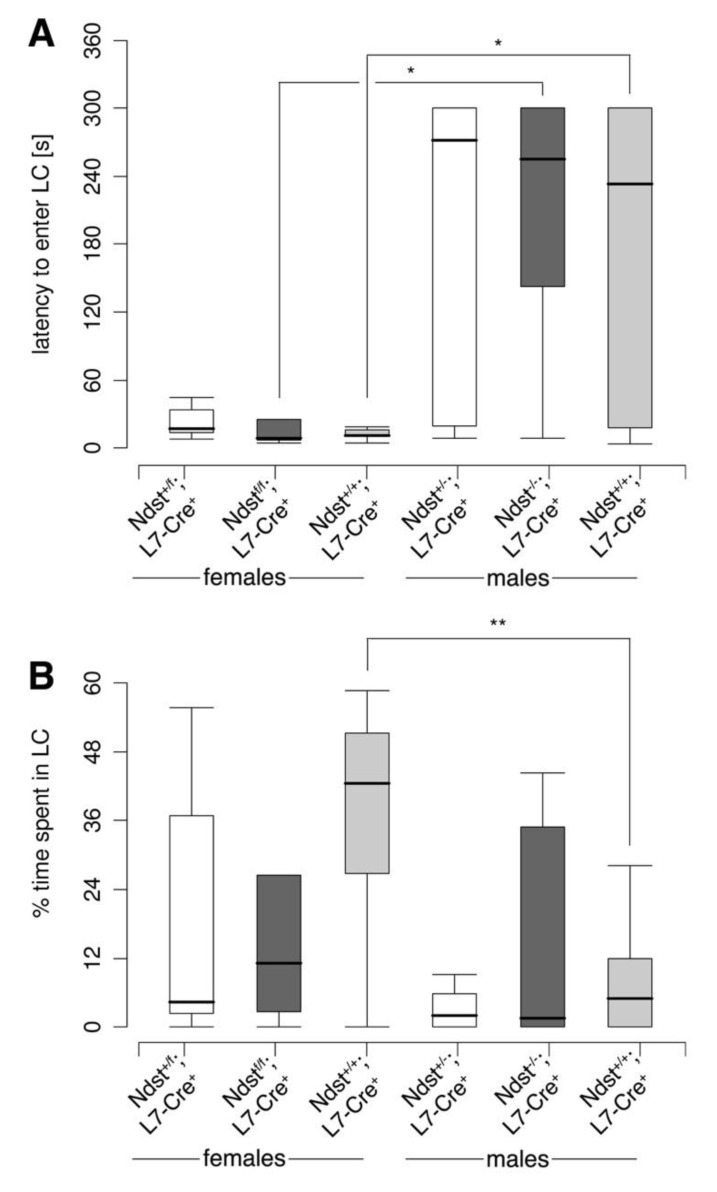
Dark–light test. (**A**) Latency to enter the light compartment. Exact Wilcoxon rank sum test, two tailed; * = *p* ≤ 0.05, T = 0.05 ≤ *p* ≤ 0.1; f *Ndst1^+/f^;L7-Cre^+^ n* = 11, f *Ndst1^f/f^;L7-Cre^+^ n* = 13, f *Ndst1^+/+^;L7-Cre^+^ n* = 9, m *Ndst1^+/f^;L7-Cre^+^ n* = 13, m *Ndst1^f/f^;L7-Cre^+^ n* = 9, m *Ndst1^+/+^;L7-Cre^+^ n* = 12; *Ndst1^f/f^; f L7-Cre^+^* versus m *Ndst1^f/f^;L7-Cre^+^*: W = 25, *p* = 0.02338 (*); f *Ndst1^+/+^;L7-Cre^+^* versus m *Ndst1^+/+^;L7-Cre^+^*: W = 26.5; *p* = 0.04883 (*). (**B**) Percentage of time spent in the light compartment. Exact Wilcoxon rank sum test, two tailed; ** = *p* ≤ 0.01, T = 0,05 ≤ *p* ≤ 0.1; f *Ndst1^+/f^;L7-Cre^+^ n* = 11, f *Ndst1^f/f^;L7-Cre^+^ n* = 13, f *Ndst1^+/+^;L7-Cre^+^ n* = 9, m *Ndst1^+/f^;L7-Cre^+^ n* = 13, m *Ndst1^f/f^;L7-Cre^+^ n* = 9, m *Ndst1^+/+^;L7-Cre^+^ n* = 12; f *Ndst1^+/+^;L7-Cre^+^* versus m *Ndst1^+/+^;L7-Cre^+^*: W = 90.5, *p* = 0.007223 (**).

We first conducted the dark–light test as an unforced measure of anxiety (Figure 6). Mice were placed into a dark compartment with the opportunity to enter and explore an illuminated compartment. The latency to enter the light compartment and the percentage of entries into the light compartment were measured. As shown in Figure 6A, *Ndst1^f/f^;L7-Cre^+^* mutant males showed enhanced latency to enter the light compartment if compared to *Ndst1^f/f^;L7-Cre^+^* mutant females (Exact Wilcoxon rank sum test, W = 25, *p* = 0.02338 (*)). We also found a significant difference between *Ndst1^+/+^;L7-Cre^+^* females and males, showing that females entered the light compartment significantly earlier than the male mice (Exact Wilcoxon rank sum test, W = 26.5, *p* = 0.04883 (*)). However, no genotype differences were determined, indicating normal anxiety in this experimental setting. Likewise, as shown in Figure 6B, *Ndst1^+/+^;L7-Cre^+^* females and males differed significantly in time spent in the light compartment. Here, females spent significantly more time in the light compartment than male mice (Exact Wilcoxon rank sum test, W = 90.5, *p* = 0.007223 (**)). However, genotype differences were not detected.

**Figure 7:**
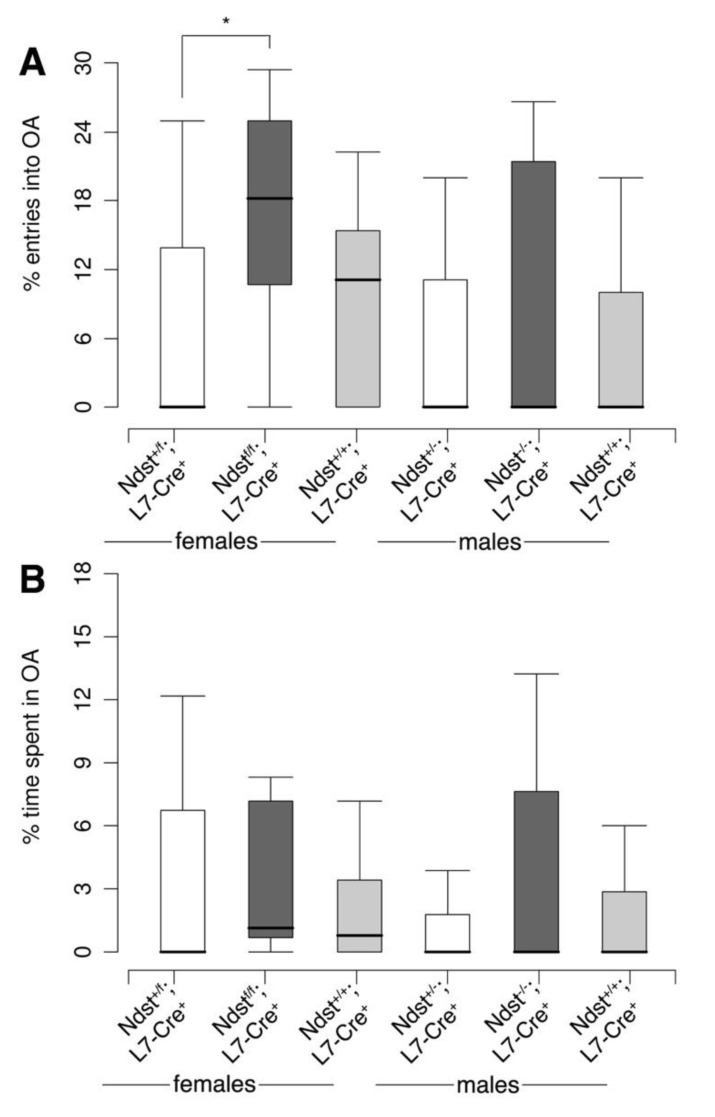
Elevated plus maze. (**A**) Percentage of entries into the open arms. Exact Wilcoxon rank sum test, two tailed; * = *p* ≤ 0.05, T = 0,05 ≤ *p* ≤ 0.1; f *Ndst1^+/f^;L7-Cre^+^ n* = 11, f *Ndst1^f/f^;L7-Cre^+^ n* = 13, f *Ndst1^+/+^;L7-Cre^+^ n* = 9, *m Ndst1^+/f^;L7-Cre^+^ n* = 13, m *Ndst1^f/f^;L7-Cre^+^ n* = 9, m *Ndst1^+/+^;L7-Cre^+^ n* = 12; f *Ndst1^+/f^;L7-Cre^+^* versus f *Ndst1^f/f^;L7-Cre^+^*: W = 38, *p* = 0.0442 (*). (**B**) Percentage of time spent in the open arms. The figure compares the percentage of time spent in the open arms of the maze. Exact Wilcoxon rank sum test, two tailed; f *Ndst1^+/f^;L7-Cre^+^ n* = 11, f *Ndst1^f/f^;L7-Cre^+^ n* = 13, f *Ndst1^+/+^;L7-Cre^+^ n* = 9, m *Ndst1^+/f^;L7-Cre^+^ n* = 13, m *Ndst1^f/f^;L7-Cre^+^ n* = 9, m *Ndst1^+/+^;L7-Cre^+^ n* = 12.

Mice were next confronted with a forced, potentially dangerous situation in the elevated plus maze (Figure 7). Mice were placed in the brightly illuminated, elevated maze with the choice to enter open or enclosed (protective) arms. Both the percentage of entries in the open arms (Figure 7A) and the percentage of time spent in the open arms (Figure 7B) were determined. We found that *Ndst1^f/f^;L7-Cre^+^* females entered the open arms significantly more often than *Ndst1^+/f^;L7-Cre^+^* heterozygous females (Exact Wilcoxon rank sum test, W = 38, *p* = 0.0442 (*)); however, no differences were found among male mice or female *Ndst1^f/f^;L7-Cre^+^* and *Ndst1^+/+^;L7-Cre^+^* mice. We also did not observe any significant differences in the time spent in the open arms between genotypes or between genders.

**Figure 8:**
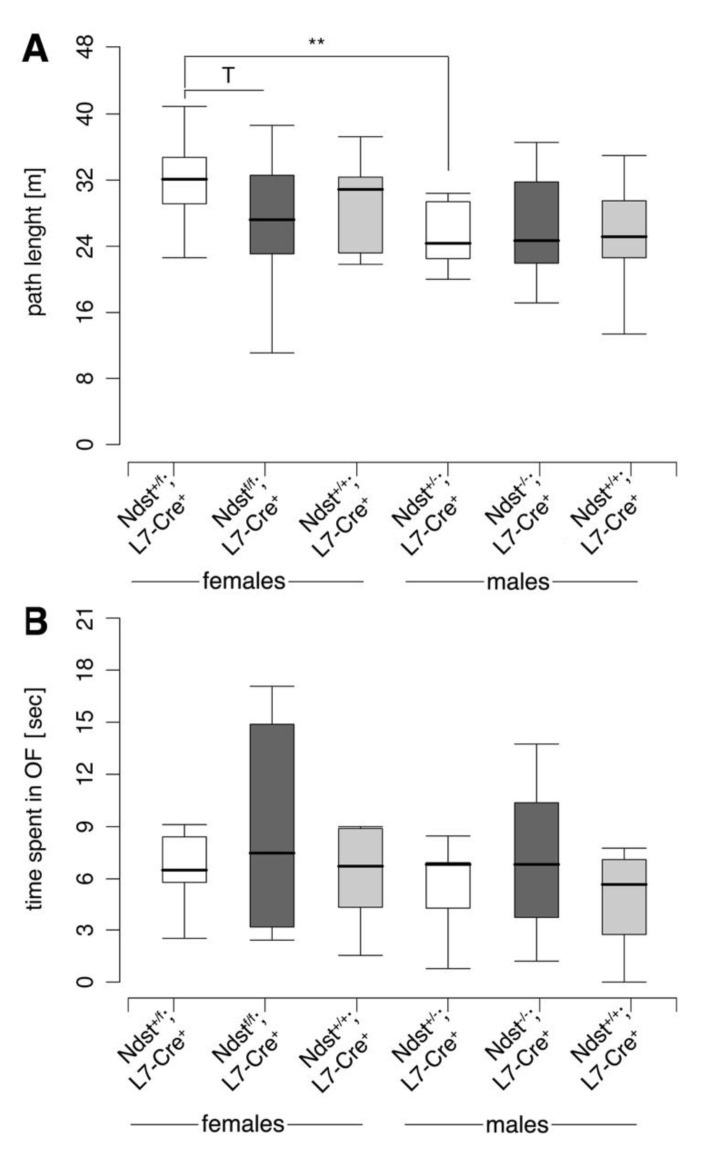
Open field test. (**A**) Path length in the open field. Exact Wilcoxon rank sum test, two tailed; ** = *p* ≤ 0.01, T = 0.05 ≤ *p* ≤ 0.1; *f Ndst1^+/f^;L7-Cre^+^ n* = 11, f *Ndst1^f/f^;L7-Cre^+^ n* = 13, f *Ndst1^+/+^;L7-Cre^+^ n* = 9, m *Ndst1^+/f^;L7-Cre^+^ n* = 13, m *Ndst1^f/f^;L7-Cre^+^ n* = 9, m *Ndst1^+/+^;L7-Cre^+^ n* = 12; f *Ndst1^+/f^;L7-Cre^+^* versus m *Ndst1^+/f^;L7-Cre^+^*: W = 118, *p* = 0.005943 (**). f *Ndst1^+/f^;L7-Cre^+^* versus f *Ndst1^f/f^;L7-Cre^+^*: W = 100.5, *p* = 0.096 (T). (**B**) Percentage of time spent in the center field. Exact Wilcoxon rank sum test, two tailed; f *Ndst1^+/f^;L7-Cre^+^ n* = 11, f *Ndst1^f/f^;L7-Cre^+^ n* = 13, f *Ndst1^+/+^;L7-Cre^+^ n* = 9, m *Ndst1^+/f^;L7-Cre^+^ n* = 13, m *Ndst1^f/f^;L7-Cre^+^ n* = 9, m *Ndst1^+/+^;L7-Cre^+^ n* = 12.

The open field test represents another type of anxiety test. Here, mice are forced into a potentially threatening environment without a possibility to hide. Path length (in meters) (Figure 8A) and the percentage of time spent in center field (with 20 cm distance from the walls, Figure 8B) were measured. The time spent in the center field was then divided by the total time spent in the open field. This test detected a highly significant difference between heterozygous males and females, because females covered a significantly longer distance than male subjects of the same genotype (Exact Wilcoxon rank sum test, W = 118, *p* = 0.005943 (**)). A trend between heterozygous and *Ndst1^f/f^;L7-Cre^+^* mutant females was found, because heterozygous females covered a longer distance in the open field than *Ndst1^f/f^;L7-Cre^+^* females (Exact Wilcoxon rank sum test, W = 100.5, *p* = 0.096 (T)). However, again, no significant genotype differences could be detected.

### 3.5. Reproductive Behavior in Compound Mutant Mice

*Ndst1* is strongly and *Ndst2* is weakly expressed in Purkinje neurons (Figure 1 and Figure 5) as well as in other cell types (Figure 5), and Ndst2 is known to compensate for loss of Ndst1 function in various systems [50]. Thus, in order to test whether normal behavior of mutant mice can be explained by compensation of Ndst1 loss of function by Ndst2, we generated *Ndst1^+/f^;Ndst2^−/−^;L7-Cre^+^* compound mutant mice. We then attempted to interbreed these mice in order to generate *Ndst1^f/f^;Ndst2^−/−^;L7-Cre^+^* transgenic mice. However, only 50% (*n* = 3 of 6) *Ndst1^+/f^;Ndst2^−/−^;L7-Cre^+^* females gave birth to pups (*n* = 14) that all died in their first days of life. This was unrelated to the pups genotype, because breeding of *Ndst1^+/f^;Ndst2^−/−^;L7-Cre^+^* females with wild-type males also did not yield surviving offspring. However, seven *Ndst1^f/f^;Ndst2^−/−^;L7-Cre^+^* mice could be successfully generated from inbred *Ndst1^f/f^;Ndst2^+/−^;L7-Cre^+^* mice. Yet, none of the obtained female *Ndst1^f/f^;Ndst2^−/−^;L7-Cre^+^* mice (*n* = 4) ever gave birth to pups and they never showed visible signs of pregnancy, even if bred to wild-type male mice. In contrast, all obtained *Ndst1^f/f^;Ndst2^−/−^;L7-Cre^+^* males (*n* = 3) were fertile, and mothers with other genotypes (*Ndst1^+/+^;Ndst2^−/−^;L7-Cre^+^* or *Ndst1^f/f^;Ndst2^+/+^;L7-Cre^+^*) also gave birth to surviving pups. Altogether, we observed that breeding of all four *Ndst1^f/f^;Ndst2^−/−^;L7-Cre^+^* females and seven *Ndst1^+/f^;Ndst2^−/−^;L7-Cre^+^* females resulted in only one surviving litter, whereas ten *Ndst1^+/+^;Ndst2^−/−^;L7-Cre^+^* or *Ndst1^f/f^;Ndst2^+/+^;L7-Cre^+^* females gave birth to seven surviving litters in the same time period. Chi_2_ analysis showed that the observed differences in reproduction between *Ndst1^f/f^;Ndst2^−/−^;L7-Cre^+^* or *Ndst1^+/f^;Ndst2^−/−^;L7-Cre^+^* female mice and control *Ndst1^+/+^;Ndst2^−/−^;L7-Cre^+^* or *Ndst1^f/f^;Ndst2^+/+^;L7-Cre^+^* females were significant (*p* < 0.01). The underlying reasons for the impaired capacity of females to reproduce remained unclear, and thus prevented further behavioral testing of sufficient numbers of *Ndst1^f/f^;Ndst2^−/−^;L7-Cre^+^ mice*. Yet, the analysis of the seven obtained *Ndst1^f/f^;Ndst2^−/−^;L7-Cre^+^* mice did not reveal any significant changes in viability, gait, or cerebellar foliation.

## 4. Discussion

HS regulates embryonic development by simultaneously binding numerous growth factors and morphogens, including but not restricted to Fgfs and Shh [9,13,48,51]. Shh as well as Shh receptors Patched and Smoothened are expressed in cerebellar Purkinje cells [26,27], and Shh is known to play crucial roles in prenatal [52] and postnatal [53,54,55,56] development of the cerebellum. Therefore, and because of the vast number of HS-binding, soluble factors expressed in the adult brain, HS is likely important for unimpaired physiology of differentiated brain tissues as well. Indeed, in the past years, the expression of HS-proteoglycans was shown to control behavior and memory in mice [14,15,16,18,57]. Moreover, altered Ndst3 expression has been linked to shizophrenia and bipolar disorder [22], which may be linked to altered HS-dependent neuregulin-1 distribution and signaling [58]. Together, these established HS functions in the adult nervous system prompted us to investigate the relevance of Ndst1, a key HS-modifier essential for embryonic development [9], in the adult murine brain. To this end, we deleted Ndst1 function by L7/pcp-Cre expression in Purkinje neurons, and expected morphological and functional consequences based on the known essential role of these cells in motor coordination.

Contrary to our expectations, however, we found that cerebellar morphology and function in adult *Ndst1^f/f^;L7-Cre^+^* mice were unchanged, indicating a non-essential role for cell-autonomous Ndst1 expression in Purkinje cells. This may be explained by compensation of Ndst1 activity by other expressed Ndst family members, as has been observed for embryonic stem cells deficient in Ndst1 and Ndst2 [59]. One model of HS biosynthesis suggests that several of the biosynthetic enzymes are present in a multienzyme complex termed the GAGosome [60], and the GAGosome could vary in composition dependent on the HS-expressing cell type or tissue. Lack of Ndst1 expression may thus result in the altered composition of the GAGosome and functional compensation by another Ndst isoenzyme. Indeed, this possibility is supported by the observation that female mice heterozygous or homozygous for floxed *Ndst1* on a systemic Ndst2-deficient background showed strongly reduced reproductive behavior, whereas *Ndst1^f/f^;Ndst2^+/+^;L7-Cre^+^* (only Ndst1-deficient) and *Ndst1^+/+^;Ndst2^−/−^;L7-Cre^+^* (only Ndst2-deficient) females were fertile. Remarkably, the observed insufficiency of one floxed Ndst1 allele in the absence of Ndst2 gene function (in *Ndst^+/f^;Ndst2^−/−^;L7-Cre^+^* mice) seems at odds with the previous observation that mice carrying systemic deletions of Ndst1 and Ndst2 (Ndst1^+/−^;Ndst2^−/−^) are viable and fertile [50]. Although we do not know the reason for this discrepancy, we note that in the conditional Ndst1 mutant only the exon 2 (the first coding exon) is targeted, but the promotor, the transcription start site and much of the 5′untranslated region remained intact [9]. In contrast, the systemic Ndst1 mutant replaced a 6.8 kb genomic fragment containing exon 2 and much of the upstream sequence with a neomycin cassette [61]. We thus suggest that the presence of two genomic sites for transcription factors and other regulators in heterozygous floxed mice, only one of which would yield functional protein after Cre expression, may functionally differ from the presence of only one promotor and 5′untranslated region in the systemic mutant. Along the same line, the observed fertility variation between *Ndst1^+/f^;Ndst2^−/−^;L7-Cre^+^* female mice and *Ndst1^f/f^;Ndst2^+/−^;L7-Cre^+^* female mice may be due to different Ndst gene dosage (insufficient Ndst1 expression from the one functional allele if compared to Ndst2). Alternatively, these differences can be explained by the inability of Ndst1 to fully compensate for the loss of Ndst2 in some L7-Cre-expressing cell types, consistent with their specific HS biosynthetic profiles [3]. Regardless of the relative Ndst activities in the process, our finding that impaired HS biosynthesis as a consequence of L7-Cre mediated Ndst1 deletion in an Ndst2-deficient background in Purkinje neurons or other cell types that express Cre from the L7 promotor (the olfactory bulb, cerebral cortex, caudate putamen, hippocampus, thalamus, midbrain and medulla oblongata) affects breeding is consistent with impaired reproduction described in the hyperspiny mouse mutant (carrying a mutation that affects Purkinje cell morphology and function) [62] and in female HS 3O-sulfotransferase 1 mutant mice [63]. Therefore, together with these reports, our findings indicate the possibility that fully sulfated HS in cell types that express Cre under L7 promotor control plays a previously unsuspected role in the regulation of female reproductive behavior. Further support for essential HS functions in normal brain physiology comes from the observations that *Ndst1* is a candidate gene for autosomal recessive intellectual disability in four unrelated families [64,65], that the *Drosophila* Ndst orthologue Sulfateless plays a role in normal social interactions and repetitive behavior in the fly [66], that Glypican 4 associates with hyperactivity and social interaction deficits in mice [67], and that a *HS 6-O sulfotransferase* gene variant associates with intellectual disability in humans [68].

Notably, we failed to observe any additional obvious problems in all seven obtained *Ndst1^f/f^;Ndst2^−/−^;L7-Cre^+^* compound mutant mice, such as in motor coordination and cerebellar architecture. In contrast, mice and tissues defective in both Ndst1 and Ndst2 function are usually strongly affected [50,59,69]. In addition to residual compensatory Ndst3 and Ndst4 activities that may explain this finding, we suggest that the rescue of impaired Purkinje cell HS biosynthesis and function may have occurred in trans by HS produced from other cell types, as has recently been described for VEGFR signaling [70]. Here, chimeric cultures of embryonic stem cells defective in either HS production or VEGFR-2 synthesis supported VEGF signaling in trans. Because cerebellar Purkinje cells constitute a single cell layer with elaborate, branched dendrites and long axons that make extensive contact with other cell types and the extracellular matrix, HS produced by other cell types in the cerebellum may have compensated for the loss of Purkinje cell HS in trans, at least to some part.

Taken together, our results show that, although Ndst1 plays crucial roles during development of the embryonic central nervous system, its cell-autonomous expression is not essential for L7-Cre expressing neurons in the adult mouse brain. Reduced reproductive capacity observed in *Ndst1/Ndst2* compound mutant mice, however, confirms the essential yet specific roles of fully sulfated HS in adult physiology.
